# Placing smoking prevalence in the context of tobacco control measures in Europe

**DOI:** 10.25646/13418

**Published:** 2025-09-24

**Authors:** Anne Starker, Dorothea Mößnang, Ronny Kuhnert

**Affiliations:** Robert Koch Institute, Department of Epidemiology and Health Monitoring, Berlin, Germany

**Keywords:** Smoking, Smoking behaviour, Tobacco consumption, Prevalence, Tobacco Control Scale, European countries, World Health Organization

## Abstract

**Background:**

The World Health Organization’s Framework Convention on Tobacco Control recommends tobacco control measures. The implementation of these measures in Europe is assessed using the Tobacco Control Scale. The aim of this analysis is to examine smoking behaviour in European countries in the context of national measures to curb tobacco consumption.

**Methods:**

In addition to analysing current tobacco consumption, the relationship between the 2019 Tobacco Control Scale results, including policy areas, and smoking prevalence in 29 European countries was examined based on data from the third wave of the European Health Interview Survey (EHIS 3). This was visualised using scatter plots and analysed using Pearson’s correlation coefficients.

**Results:**

On average, 24.4 % of adults in Europe smoke, with substantial differences between countries. There are also some clear differences between the sexes, with a higher proportion of men than women smoking in all countries studied. A higher overall score on the Tobacco Control Scale is associated with a lower smoking prevalence among both women and men. Negative correlations are also evident between smoking prevalence and the policy areas of smoking cessation support and advertising bans.

**Conclusions:**

Consistent implementation of tobacco control measures is associated with lower smoking prevalence in Europe. This highlights the importance of comprehensive tobacco control strategies to reduce tobacco consumption.

## 1. Introduction

Despite the global decline in smoking prevalence over the past two decades, the World Health Organization (WHO) target of a 30 % reduction by 2025 compared to 2010 will only be achieved in two WHO regions (Africa and South-East Asia) [1]. With a forecast decline of 17 %, Europe is lagging far behind the other regions. According to data from the Global Burden of Disease (GBD) study, approximately 7.3 million people worldwide died from tobacco related causes in 2021, including 1.1 million in the WHO European Region [2]. In most European countries, 10 % to 20 % of the burden of disease due to death and health impairments is attributable to smoking [3]. Tobacco consumption therefore remains one of the most important single risk factors for the development of non-communicable diseases.

In response to the global tobacco epidemic, the WHO Framework Convention on Tobacco Control (FCTC) was developed with the stated aim of protecting the population from the health, social, environmental and economic consequences of tobacco consumption and passive smoking [4]. It is an international treaty that was adopted by the 56th World Health Assembly and entered into force on 27 February 2005. The FCTC sets out specific measures to reduce both the demand for and supply of tobacco products, such as price and tax measures or measures to restrict access to tobacco products by minors.

In order to systematically quantify the implementation of measures to reduce tobacco consumption in accordance with the FCTC in European countries, the www.tobaccocontrolscale.org was developed, which is published every three years [5], most recently for the year 2021. Initiative funding for this was provided in 2004 by the European Network for Smoking Prevention with the support of the European Commission. The Tobacco Control Scale is currently partly funded by the European Commission through Smoke Free Partnership, a European association of non-governmental organisations that work to implement the FCTC and achieve a smoke-free future in Europe [6]. The scale is used to assess the implementation of priority areas of policy areas, such as increasing cigarette prices, comprehensive bans on advertising and promotion, and measures to support smoking cessation. A higher score for the different policy areas and the overall score on the Tobacco Control Scale indicates that a country has implemented more and stricter tobacco control measures. The weighting of the different policy areas was determined by an international panel of experts (see 2.3 for details).

Article 20 of the FCTC recommends collecting data on the prevalence, patterns, determinants and consequences of tobacco consumption and exposure to tobacco smoke [4]. The aim is to ensure that countries collect, analyse and share scientific data on tobacco consumption, health effects and the effectiveness of tobacco control measures. This requirement is met by the European Health Interview Survey (EHIS), which provides data on the prevalence and determinants of smoking in European countries on a regular basis, every six years since 2019. The third wave of the EHIS (EHIS 3, 2019) provides comparable data across Europe, which can be used to describe differences in the prevalence of smoking.

Previous studies have analysed the relationship between Tobacco Control Scale scores and the prevalence of smoking among adults. Two studies from 2008 and 2016 used regression analysis to examine the impact of country-specific tobacco control measures on smoking cessation rates among different educational groups [7, 8]. According to these studies, the country-specific score on the Tobacco Control Scale was positively correlated with smoking cessation rates across all age and gender groups, as determined by national health surveys and the Eurobarometer survey, which involved approximately 1,000 respondents per country. Other studies from 2010 and 2016 used correlation to examine the association between the Tobacco Control Scale and the prevalence of tobacco consumption in Europe [9, 10]. These studies showed that countries with higher scores on the Tobacco Control Scale had lower smoking prevalence rates [9], higher smoking cessation rates and greater relative declines in smoking prevalence [10].

**Key messages**

► The implementation of tobacco control measures in European countries can be compared using the Tobacco Control Scale.
► EHIS 3 provides current smoking prevalence rates for European countries, ranging from 12.8 % in Sweden to 36.6 % in Bulgaria.
► A higher overall score on the 2019 Tobacco Control Scale is associated with a lower smoking prevalence.
► In the individual areas of action, higher scores for support in quitting smoking and advertising bans are associated with low smoking prevalence.
► The results underscore the importance of more consistent implementation of tobacco control measures.

This study aims to analyse the prevalence of tobacco smoking in European countries in 2019 and to examine whether a higher overall score on the Tobacco Control Scale as well as in different policy areas is associated with lower smoking prevalence. For the first time, the latest data from EHIS 3 with a large sample and the results of the 2019 Tobacco Control Scale will be used for this purpose.

## 2. Methods

### 2.1 Sample design and study implementation of EHIS 3

EHIS 3 was conducted in all Member States of the European Union (EU) as well as Iceland, Norway, Serbia, Albania and Turkey. The target population comprises people aged 15 and over who live in private households. The survey consists of four modules on the topics of health status, health care, health determinants and socioeconomic situation.

Data collection for EHIS 3 took place in 2019. The participating countries selected nationally representative samples based on population registers, censuses, housing registers or other sources. Some countries were granted an exemption regarding the data collection period, so that data is available for the period from January 2018 to September 2020. According to the EHIS implementing regulation, the data collection period should be spread over at least three months, including at least one month in the autumn season (September to November). There are recommendations and guidelines for the methodology and implementation of the survey, which ensure a high degree of harmonisation of results between Member States [11]. A quality report contains detailed information on the methodological approach of the individual countries [12]. With the exception of France, Albania and Turkey, the EHIS 3 data are available for analyses from all participating countries. For research purposes such as this study, anonymised data at participant level (microdata) can be requested from the Statistical Office of the European Union (Eurostat) [13]. Results based on aggregated data are available on the Eurostat website [14].

### 2.2 Selected EHIS 3 indicators

#### Current tobacco smoking

Current tobacco smoking behaviour was recorded using the following question: ‘Do you smoke tobacco products including heated tobacco products? Please exclude electronic cigarettes or similar products’. The response categories were: ‘Yes, daily’, ‘Yes, occasionally’, ‘No, not anymore’ and ‘I have never smoked’. Based on the response categories, current tobacco smoking is defined as daily or occasional smoking of tobacco products, including heated tobacco products.

#### Gender

Participants were asked about their biological sex (female or male).

### 2.3 Assessment of tobacco control activities using the Tobacco Control Scale

The Tobacco Control Scale quantifies the implementation of measures to reduce tobacco consumption in 36 European countries [15]. The scale is based on six policy areas described by the World Bank [16] and in the FCTC [4] that should be implemented as a priority to reduce tobacco consumption. These include: (1) price increases through higher taxes on cigarettes and other tobacco products, (2) banning/restricting smoking in public places and workplaces, (3) better consumer information, including public information campaigns, media coverage and publication of research findings, (4) comprehensive bans on advertising and promotion of all tobacco products, logos and brand names, (5) large, direct health warnings on cigarette packets and other tobacco products, (6) measures to support dependent smokers in quitting, including better access to medication. The Tobacco Control Scale also assessed measures against the illicit tobacco trade (7), measures against tobacco industry influence (8), and ratification of the WHO FCTC (9).

The degree to which different measures are implemented within the policy areas is assessed on a points basis [5], with a maximum score of 100 points. One point is deducted for non-ratification of the WHO FCTC (Table 1). The criteria for awarding points and their distribution were developed by Luk Joossens and Martin Raw, two internationally renowned experts in tobacco control policy, first published in 2006 [5], and regularly updated and adjusted. The point score can be used to assess the overall tobacco control efforts of countries and identify areas for improvement in the different policy areas [5]. Information on countries’ tobacco control budgets and tobacco cessation treatment measures is taken from a standardised questionnaire completed by national experts. Furthermore, information from the WHO and the EU on cigarette prices, advertising bans, smoking bans and Eurobarometer results on the enforcement of non-smoker protection laws are also included. The weighting of the eight policy areas was developed by a group of experts from the European Network for Smoking Prevention (see Table 1 for the 2019 Tobacco Control Scale scoring system). It is based in part on subjective assessments by the experts, as reliable data on causal effectiveness is not available for all policy areas. The Tobacco Control Scale is published every three years. The results of the 2019 Tobacco Control Scale were used for the present analyses [17].

**Infobox Web portal of Federal Health Reporting**

The website www.gbe.rki.de/EN of Federal Health Reporting at the Robert Koch Institute (RKI) provides reliable information on the health situation of the population in Germany: timely, transparent and easily accessible. The focus is on non-communicable diseases such as diabetes mellitus, cardiovascular diseases, cancer and mental disorders. It also presents factors that have an influence on health, such as health behaviour or social determinants. In addition, the web portal provides information on health care and contextual factors, such as food taxation or tobacco control measures, which also influence the health of the population.

The website currently includes over 60 indicators from health monitoring at the RKI and other data sources, which are interactively visualised and contextualised in short texts. The data is published as open data on GitHub and Zenodo. In addition, the website provides access to all RKI publications that are related to the topics on the website. The content is continuously expanded.

Further information on the topic of this article can be found on the web portal at

www.gbe.rki.de/smoking

www.gbe.rki.de/tobacco-control

### 2.4 Selected policy areas from the 2019 Tobacco Control Scale

For the present evaluations, the scores of the countries were included for those policy areas (brief description) of the 2019 Tobacco Control Scale for which data was available for all countries [17] and which had carried out EHIS 3. These are the following six policy areas for 29 countries: price, public consumption bans, advertising bans, health warnings, support for quitting smoking and combating illegal tobacco trade (Annex Table 1).

### 2.5 Statistical methods

#### Tobacco smoking prevalence

The EHIS 3 dataset includes data from 311,385 participants aged 15 and older from 29 European countries. For the statistical analyses, individual data from EHIS 3 was used for those who completed the questionnaire themselves (n = 296,841). Data from the age of 20 onwards is available for Malta and Iceland (Annex Table 2). Weighted EHIS 3 microdata was used to ensure that each country was represented proportionally to its population size.

Descriptive analyses were performed by calculating prevalence (relative frequencies) with 95 % confidence intervals (95 % CI). To account for age-related differences between countries and enable direct comparisons of country prevalence estimates, the calculations were performed using direct age standardisation. The age structures and gender distributions of the country samples were adjusted to the 2013 European standard population [18].

#### Association between smoking behaviour and tobacco control measures

The association between total score on the Tobacco Control Scale and the different policy areas, and current tobacco smoking is described using correlation coefficients and visualised with scatter plots. To quantify the association, the weighted Pearson correlation coefficient (r_P_) is first calculated. The calculation is based on the previously determined smoking prevalence of each country and their results in the 2019 European Tobacco Control Scale. To avoid bias due to different sample sizes, the countries were weighted according to their population size.

Standard errors and 95 % confidence intervals were calculated using the bootstrap method and Fisher’s Z-transformation to estimate the uncertainty of the correlation coefficient. The Pearson correlation coefficient was chosen for the correlation analysis because an approximate linear relationship was assumed, and the analysis was based on the point score of the Tobacco Control Scale. The calculated correlations do not permit any conclusions to be drawn regarding the effectiveness of particular measures. Furthermore, the correlations between the different policy areas of the Tobacco Control Scale and smoking prevalence cannot be interpreted as independent effects, because the policy areas may overlap in terms of content and have similar mechanisms of action. All analyses were performed using R 4.4.1/R Studio 2024.04.0.735 and Stata Version 17.0.

## 3. Results

On average in Europe, 24.4 % of adults currently smoke tobacco products. There are considerable differences in the prevalence of current tobacco smoking: the lowest prevalence is in Sweden, at 12.8 % and the highest is in Bulgaria, at 36.2 %. In Serbia, too, the smoking prevalence is over 30 %. In Germany, the smoking prevalence is 28.7 %, which is above the European average. There are some considerable gender differences (Annex Table 3), with more men than women smoking in all countries considered.

The scores on the Tobacco Control Scale show some parallels with smoking prevalence. Ireland achieved the best rating on the scale in 2019 with 73 points, which can be attributed to comprehensive tobacco control measures such as strict smoking bans and far-reaching advertising bans. The overall smoking prevalence in Ireland is 17.7 %, with 15.7 % among women and 19.8 % among men. This makes Ireland the country with the fifth lowest smoking prevalence in the EU-29 countries in 2019. Iceland and Norway scored 70 and 66 points, respectively, on the 2019 Tobacco Control Scale, placing them in second and third place. Here too, smoking prevalence among the general population is low: 13.8 % of the population smoke in Iceland and 17.9 % in Norway. Among the countries with a score of over 50 points on the Tobacco Control Scale, Slovenia, Belgium, the Netherlands, Italy, Sweden and Portugal have smoking prevalence rates below the European average. Germany has the lowest score on the 2019 Tobacco Control Scale with 40 points. Tobacco control measures here are comparatively weak, with fewer comprehensive smoking bans and less stringent regulations on tobacco advertising. With a smoking prevalence of 28.7 %, Germany has the fourth highest rate among the 29 European countries. Luxembourg and Serbia have the lowest scores on the 2019 Tobacco Control Scale after Germany. However, the smoking prevalence in Luxembourg is low by European standards, at 17.2 %. In Serbia, the smoking prevalence of the total population is 31.5 %, which is the second highest among the countries compared.

**Figure 1:**
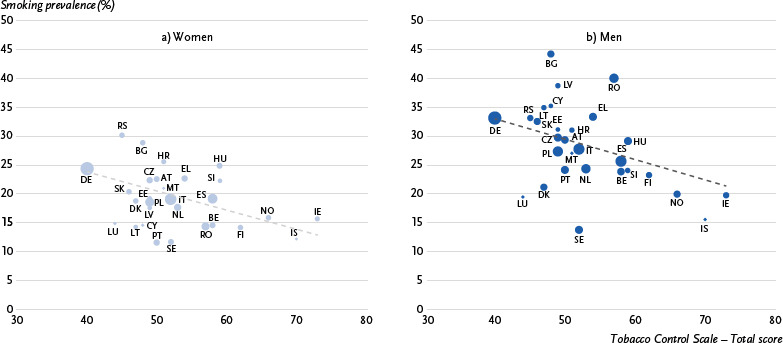
Correlation of estimated smoking prevalence in European countries with the total score on the 2019 Tobacco Control Scale for women (1a) and men (1b), weighted by population size. Source: EHIS 3 (2019), Tobacco Control Scale 2019 AT = Austria, BE = Belgium, BG = Bulgaria, CY = Cyprus, CZ = Czech Republic, DE = Germany, DK = Denmark, EE = Estonia, EL = Greece, ES = Spain, FI = Finland, HR = Croatia, HU = Hungary, IE = Ireland, IS = Iceland, IT = Italy, LT = Lithuania, LU = Luxembourg, LV = Latvia, MT = Malta, NL = Netherlands, NO = Norway, PL = Poland, PT = Portugal, RO = Romania, RS = Serbia, SE = Sweden, SI = Slovenia, SK = Slovakia

The correlation coefficients confirm these observations. There is an inverse relationship between the total score of the Tobacco Control Scale and the prevalence of smoking in the 29 European countries (r_P_ = - 0.58) (Table 2). Higher scores on the Tobacco Control Scale are therefore associated with lower proportions of smokers. Similar results are found for women and men: for women, the Pearson correlation coefficient is r_P_ = - 0.58 and for men it is r_P_ = - 0.45 (Figure 1a and Figure 1b).

The individual policy areas of the Tobacco Control Scale – price, public consumption bans, advertising bans, health warnings and support for quitting smoking – are also inversely related to smoking prevalence. However, only the policy areas of support for quitting smoking (r_P_ = - 0.53) and advertising bans (r_P_ = - 0.56) show statistically significant correlations. In the gender-separated analysis, there is a significant correlation between smoking prevalence and the two policy areas on the Tobacco Control Scale (Table 2). The correlations identified show that some policy areas of the Tobacco Control Scale are more strongly associated with smoking prevalence than others. However, these results are purely descriptive and do not allow any conclusions to be drawn about their effectiveness.

## 4. Discussion

### 4.1 Brief summary

On average, 24.4 % of adults in Europe smoke, with smoking prevalence varying greatly between countries – from 12.8 % in Sweden to 36.2 % in Bulgaria. In all countries studied, more men than women smoke.

The results show a negative correlation between a country’s Tobacco Control Scale score and its smoking prevalence. Countries with a higher overall Tobacco Control Scale score have lower smoking prevalences than countries with a lower score. Of the policy areas considered by the Tobacco Control Scale, support for quitting smoking and advertising bans are associated with lower tobacco smoking prevalence at the country level. The results enable a current data-based classification of the degree of implementation of tobacco control measures with regard to smoking prevalence in 29 European countries.

### 4.2 Classification

Previous studies have examined how the results of the European Tobacco Control Scale relate to adult smoking behaviour in different European countries. These show a negative correlation between the result of the Tobacco Control Scale (overall score) and smoking prevalence: countries with a higher overall score on the Tobacco Control Scale had a lower smoking prevalence than those with a lower overall score [9, 10, 19]. This is consistent with the results of the present analysis. In the different policy areas, higher scores for public consumption bans and health warnings were significantly correlated with lower smoking prevalence [10].

There are gender differences in that statistically significant correlations exist for men in relation to public consumption bans, spending on public information campaigns, health warnings and support for quitting smoking, whereas for women this is only the case for public consumption bans [10].

An older study found a significant correlation for advertising bans and health warnings [9]. This is consistent with our findings, according to which all policy areas except for combating illegal tobacco trade were negatively associated with smoking prevalence.

The present finding that price and support for quitting smoking are significantly associated with lower smoking prevalence is supported by a review with meta-analysis that identified individual counselling by healthcare professionals and guaranteed financial incentives as the most evidence-based measures for promoting smoking cessation [20]. Other studies also show that price increases are significantly associated with a decline in smoking [19, 21].

A study from Germany showed that the low acceptance of evidence-based methods and the lack of cost coverage by statutory health insurance funds are particular barriers to successful tobacco cessation [22]. This structural barrier particularly affects socioeconomically disadvantaged groups and thus contributes to the exacerbation of health inequalities.

Results from Ireland, the country with the top position on the Tobacco Control Scale, show that tobacco control measures in the areas of price and bans on consumption in public places, which were implemented between 1998 and 2016, contributed most to the reduction in smoking prevalence in 2016, followed by support for smoking cessation, health warnings and advertising bans [23].

In 2025, the fourth wave of the EHIS will be conducted in European Member States. This will allow for a review and assessment of the medium- to long-term relationship between tobacco control measures and smoking prevalence in Europe.

### 4.3 Strengths and limitations

The large sample of over 296,000 participants in the third wave of the EHIS along with the participation of all European countries, enables representation of the European population as a whole. The third EHIS wave provides the most up-to-date Europe-wide data on smoking behaviour at country level. Based on current knowledge, these data are being examined for the first time in terms of smoking behaviour and tobacco control measures.

Due to the aggregated data and the unclear temporal sequence between tobacco control measures and smoking prevalence, no causal relationship can be inferred. Therefore, the correlations observed should be interpreted as associations, and no conclusions can be drawn about a cause-and-effect relationship. Furthermore, the analysis is based on smoking prevalence data from a limited number of 29 countries, which reduces the statistical significance for determining robust correlations. This is particularly reflected in the sometimes wide confidence intervals of the correlation coefficients. In addition, the low range of variation in some policy areas of the Tobacco Control Scale (e.g. combating illegal tobacco trade with a maximum of three points to be achieved or health warnings with a maximum of ten points to be achieved) further limits the meaningfulness of the calculated correlations.

Despite these limitations, the observed directions of the correlations provide valuable insights into the possible mechanisms of action of tobacco control policies at the population level. These results can serve as a starting point for further analyses that can be interpreted causally. Suitable methods for this would be, for example, quasi-experimental analyses, such as interrupted time series regression or difference-in-differences analyses, in countries that have introduced tobacco control measures at different times.

Further limitations are partly attributable to the EHIS database: when collecting self-reported data on behavioural risk factors, there is a general risk of social desirability and biased reporting. This can lead to an underestimation of the actual prevalence of smoking [24]. This study only considers the consumption of tobacco products (tobacco cigarettes and tobacco heaters), but not the consumption of e-cigarettes or water pipes, which are also considered harmful to health due to their nicotine and toxin content (for an overview, see [25–27]). Future studies should take these into account due to the increase in consumption of these products [28]. In addition, successful smoking cessation is also relevant for classifying the effectiveness of tobacco control measures. This health behaviour needs to be analysed in further studies.

Other factors influencing smoking, such as prosperity [29], social acceptance of smoking or social affiliation [30, 31], were not included in this study for contextualisation of smoking prevalence. As these factors vary between countries, taking them into account could provide further insights into the interaction of individual and structural factors on smoking prevalence.

It should also be noted critically that not all policy areas of the 2019 Tobacco Control Scale could be included in the analyses, but only those for which data was available for all countries that conducted EHIS 3. For this reason, the policy areas of spending on public information campaigns, tobacco industry interference and non-ratification of the WHO FCTC are not examined in relation to smoking prevalence.

The Tobacco Control Scale developed by Joossens and Raw in 2004 is a useful and pragmatic tool for comparing national tobacco control measures. However, rather than being based on a standardised assessment process, the assessment of individual policy areas is based on expert assessments and weightings, for which the empirical evidence base is not always clear. It does not replace a differentiated evaluation of the different measures. Given the changing political and scientific context, it therefore seems sensible to further develop the scale in a transparent and evidence-based manner in order to strengthen its informational value and relevance to current health research.

### 4.4 Conclusions

This study has shown that a consistent implementation of measures to reduce tobacco consumption, indicated by a higher overall score on the Tobacco Control Scale, is associated with a lower smoking prevalence. This result highlights the importance of implementing comprehensive measures to reduce tobacco consumption, as they positive effect on reducing smoking prevalence. Future analyses should examine how changes in Tobacco Control Scale scores over time affect the development of smoking prevalence. Associations with smoking cessation rates in the respective European countries are also an important aspect and a starting point for public health measures. Important individual influencing factors, such as gender or education, should also be considered.

## Figures and Tables

**Table 1:** Maximum points achievable for the different policy areas and total score on the 2019 Tobacco Control Scale. Source: Own table according to Joossens et al. 2020 [17]

Policy area		Brief description	Maximum points
1	Price of cigarettes^*^	Price	30
2	Smoke-free work and other public places	Public consumption bans	22
3	Spending on public information campaigns^**^	Budget	10
4	Comprehensive bans on advertising and promotion	Advertising bans	13
5	Large direct health warning labels	Health warnings	10
6	Measures to support smoking cessation	Support for quitting smoking	10
7	Illicit tobacco trade	Combating illegal tobacco trade	3
8	Tobacco industry interference	Influence of tobacco industry	2
9	No ratification of the WHO FCTC	-	-1
**Total**		-	**100**

WHO = World Health Organization, FCTC = Framework Convention on Tobacco Control

^*^Weighted average price for cigarettes in 2018 in the respective countries, taking into account the respective purchasing power standards (PPS). PPS are used to take into account the real purchasing power in different countries.

^**^Based on the average gross domestic product per capita of the respective countries expressed in PPS.

**Table 2:** Pearson correlation coefficient (r_P_) and 95 % confidence intervals (CI) between the total score of the 2019 Tobacco Control Scale, its six policy areas and smoking prevalence. Source: EHIS 3 (2019), Tobacco Control Scale 2019

Policy area	Women			Men			Total		
	r_P_	(95 % CI)	p-value	r_P_	(95 % CI)	p-value	r_P_	(95 % CI)	p-value
**Total score**	**- 0.58**	**(- 0.78 – - 0.29)**	0.008^*^	**- 0.42**	**(- 0.69 – - 0.05)**	0.020*	**- 0.48**	**(- 0.72 – - 0.15)**	0.008^*^
Price	- 0.26	(- 0.63 – 0.21)	0.205	- 0.09	(- 0.47 – 0.32)	0.688	- 0.19	(- 0.57 – 0.26)	0.432
Public consumption bans	- 0.35	(- 0.65 – 0.04)	0.068	- 0.18	(- 0.54 – 0.22)	0.328	- 0.28	(- 0.56 – 0.05)	0.090
Advertising bans	- 0.51	(- 0.76 – - 0.13)	0.006^*^	- 0.45	(- 0.71 – - 0.08)	0.004^*^	- 0.56	(- 0.71 – - 0.08)	0.002^*^
Health warnings	- 0.33	(- 0.63 – 0.05)	0.076	- 0.30	(- 0.61 – 0.08)	0.111	- 0.35	(- 0.65 – 0.04)	0.061
Support for quitting smoking	- 0.46	(- 0.71 – - 0.11)	0.012^*^	- 0.45	(- 0.69 – - 0.12)	0.006^*^	- 0.53	(- 0.76 – - 0.16)	0.002^*^
Combating illegal tobacco trade	0.20	(- 0.21 – 0.55)	0.306	0.01	(- 0.38 – 0.37)	0.948	0.01	(- 0.28 – 0.47)	0.540

^*^p < 0.05

**Annex Table 1:** Results of the 2019 Tobacco Control Scale for countries that conducted EHIS 3 and for which information was available for the respective policy areas for all countries (sorted by total score). Source: Joossens et al. 2020 [17]

Country	Price	Public consumption bans	Advertising bans	Health warnings	Support for quitting smoking	Combating illegal tobacco trade	Total Score
Ireland	18	22	13	9	8	1	73
Iceland	23	17	13	4	4	0	70
Norway	22	17	13	8	4	1	66
Finland	18	18	13	5	5	1	62
Slovenia	12	16	13	9	6	1	59
Hungary	15	21	11	5	6	1	59
Belgium	16	16	8	9	6	2	58
Spain	15	21	9	5	5	2	58
Romania	16	21	8	5	6	1	57
Greece	18	20	7	5	3	1	54
Netherlands	14	15	9	5	7	1	53
Italy	15	16	9	5	6	1	52
Sweden	14	15	9	5	7	2	52
Croatia	16	11	12	5	5	2	51
Malta	16	12	11	5	5	2	51
Austria	11	20	7	5	5	2	50
Portugal	18	11	10	5	4	2	50
Estonia	13	14	11	5	3	2	49
Latvia	14	12	10	5	4	2	49
Poland	14	11	11	5	7	1	49
Czech Republic	12	15	8	5	7	2	49
Bulgaria	15	11	11	5	5	1	48
Cyprus	15	10	11	5	5	2	48
Denmark	13	11	8	5	7	1	47
Lithuania	12	13	10	5	4	2	47
Slovakia	12	12	9	5	6	2	46
Serbia	19	11	9	1	4	1	45
Luxembourg	5	16	9	5	7	2	44
Germany	14	11	4	5	4	2	40

**Annex Table 2:** Characteristics of the study population by gender (n = 167,112 women, n = 144,273 men). Source: EHIS 3 (2019)

	Women		Men	
	n (unweighted)	% (weighted)	n (unweighted)	% (weighted)
Total	160,521	50.5	136,320	49.5
**Age**				
15 – 29 years	21,923	17.3	21,212	19.5
30 – 39 years	20,220	15.0	17,772	15.9
40 – 49 years	25,150	16.5	22,143	17.4
50 – 59 years	28,199	17.3	24,410	17.6
60 – 69 years	29,364	15.2	24,876	15.0
≥ 70 years	35,665	18.8	25,907	14.7
**Education group**				
Low	49,972	31.2	38,417	27.9
Medium	62,855	43.7	58,376	46.9
High	46,625	25.1	38,476	25.2
Missing	1,069		1,051	
**Current smoking**				
Yes	27,969	20.1	35,973	29.3
No	129,136	79.9	97,283	70.7
Missing	3,416		3,064	
**Country**				
Belgium	4,598	2.6	4,163	2.6
Bulgaria	3,930	1.8	3,301	1.8
Denmark	3,768	1.5	2,861	1.6
Germany	12,111	22.4	10,890	23.4
Estonia	2,838	0.4	2,013	0.3
Finland	3,443	1.4	2,468	1.4
Greece	4,152	2.8	3,696	2.7
Ireland	4,138	1.2	3,483	1.3
Iceland	2,043	0.1	1,838	0.1
Italy	21,041	14.4	18,327	14.0
Croatia	2,938	1.2	2,292	0.9
Latvia	3,421	0.5	2,430	0.5
Lithuania	2,860	0.8	1,976	0.7
Luxembourg	2,428	0.2	2,076	0.2
Malta	2,279	0.1	2,077	0.2
Netherlands	4,187	7.4	4,007	4.7
Norway	3,940	1.3	3,973	1.4
Austria	8,173	2.3	7,080	2.4
Poland	9,925	8.6	6,940	7.2
Portugal	8,295	2.9	6,322	2.8
Romania	8,335	5.1	7,573	5.1
Sweden	4,842	2.6	4,915	2.8
Serbia	6,420	1.8	6,043	1.8
Slovakia	3,217	1.5	2,310	1.5
Slovenia	5,353	0.5	4,410	0.6
Spain	11,229	12.1	10,080	12.5
Czech Republic	4,464	2.7	3,420	2.8
Hungary	3,031	2.7	2,572	2.6
Cyprus	3,122	0.2	2,784	0.2

**Annex Table 3:** Age-standardised prevalence of current tobacco product use in Europe. Source: EHIS 3 (2019)

Country	Total		Women		Men	
	%	95 % CI	%	95 % CI	%	95 % CI
Belgium	19.2	17.9 – 20.5	14.6	13.3 – 16.1	23.9	21.1 – 25.9
Bulgaria	36.2	34.8 – 37.6	28.9	27.3 – 30.5	44.3	42.5 – 46.2
Denmark	20.0	18.9 – 21.1	18.8	17.5 – 20.2	21.2	19.6 – 23.0
Germany	28.7	27.7 – 29.7	24.4	23.1 – 25.7	33.2	31.8 – 34.6
Estonia	24.7	23.3 – 26.0	19.0	17.4 – 20.7	31.2	29.1 – 33.4
Finland	18.2	17.0 – 19.5	14.0	12.7 – 15.5	22.9	20.9 – 25.0
Greece	28.8	27.6 – 30.1	22.4	20.8 – 24.0	35.9	34.0 – 37.7
Ireland	17.7	16.7 – 18.8	15.8	14.4 – 17.2	19.8	18.2 – 21.4
Iceland	13.8	12.4 – 15.3	12.2	10.4 – 14.1	15.6	13.5 – 18.0
Italy	23.2	22.7 – 23.8	18.9	18.2 – 19.6	28.0	27.2 – 28.8
Croatia	28.2	26.3 – 30.0	25.7	23.6 – 27.9	31.5	29.1 – 34.0
Latvia	26.8	25.5 – 28.1	17.4	16.0 – 19.0	38.4	36.3 – 40.5
Lithuania	23.5	22.3 – 24.8	14.2	12.8 – 15.6	34.8	32.7 – 37.0
Luxembourg	17.2	16.1 – 18.4	14.9	13.5 – 16.4	19.5	17.8 – 21.4
Malta	24.0	22.7 – 25.4	20.9	19.2 – 22.8	27.0	25.0 – 29.0
Netherlands	21.0	20.1 – 22.0	17.7	16.5 – 19.0	24.3	22.9 – 25.8
Norway	17.9	16.9 – 19.0	15.9	14.7 – 17.3	19.9	18.5 – 21.4
Austria	25.9	25.0 – 26.7	22.6	21.5 – 23.7	29.4	28.1 – 30.7
Poland	22.1	21.2 – 23.0	18.2	17.3 – 19.2	27.2	25.9 – 28.5
Portugal	17.5	16.5 – 18.6	11.7	10.5 – 12.9	24.2	22.5 – 26.0
Romania	26.7	25.8 – 27.7	14.4	13.4 – 15.4	40.0	38.7 – 41.4
Sweden	12.8	12.0 – 13.5	11.7	10.7 – 12.8	13.8	12.8 – 14.8
Serbia	31.5	30.2 – 32.8	30.0	28.5 – 31.5	33.2	31.6 – 34.8
Slovakia	26.3	25.0 – 27.6	20.4	18.8 – 22.0	32.7	30.6 – 34.8
Slovenia	23.0	22.1 – 24.0	22.2	21.0 – 23.4	23.9	22.6 – 25.3
Spain	22.0	21.4 – 22.8	18.9	17.9 – 19.8	25.5	24.4 – 26.6
Czech Republic	26.0	24.9 – 27.2	22.4	21.0 – 23.9	29.8	28.1 – 31.5
Hungary	27.0	25.7 – 28.2	24.9	23.2 – 26.6	29.2	27.4 – 31.1
Cyprus	24.6	23.3 – 25.9	14.6	13.3 – 16.0	35.3	33.4 – 37.2
Total	24.4	24.1 – 24.7	20.0	19.6 – 20.4	29.2	28.7 – 29.6

95 % CI = 95 % confidence interval
